# Assessment of the Interactions between Economic Growth and Industrial Wastewater Discharges Using Co-integration Analysis: A Case Study for China’s Hunan Province

**DOI:** 10.3390/ijerph8072937

**Published:** 2011-07-14

**Authors:** Qiang Xiao, Yang Gao, Dan Hu, Hong Tan, Tianxiang Wang

**Affiliations:** 1 Chongqing University of Arts and Sciences, Chongqing 402160, China; E-Mails: xiaoqiang7598@126.com (Q.X.); th1232003@yahoo.com.cn (H.T.); yxxymsx@163.com (T.W.); 2 State Key Laboratory of Urban and Regional Ecology, Center for Eco-Environmental Sciences, Chinese Academy of Sciences, Beijing 100085, China; E-Mail: hudan@rcees.ac.cn; 3 Key Laboratory of Water Cycle and Related Land Surface Processes, Institute of Geographic Sciences and Natural Resources Research, Chinese Academy of Sciences, Beijing 100101, China

**Keywords:** economic growth, industrial wastewater, error correction, vector auto-regression (VAR) model, vector error correction (VECM) model

## Abstract

We have investigated the interactions between economic growth and industrial wastewater discharge from 1978 to 2007 in China’s Hunan Province using co-integration theory and an error-correction model. Two main economic growth indicators and four representative industrial wastewater pollutants were selected to demonstrate the interaction mechanism. We found a long-term equilibrium relationship between economic growth and the discharge of industrial pollutants in wastewater between 1978 and 2007 in Hunan Province. The error-correction mechanism prevented the variable expansion for long-term relationship at quantity and scale, and the size of the error-correction parameters reflected short-term adjustments that deviate from the long-term equilibrium. When economic growth changes within a short term, the discharge of pollutants will constrain growth because the values of the parameters in the short-term equation are smaller than those in the long-term co-integrated regression equation, indicating that a remarkable long-term influence of economic growth on the discharge of industrial wastewater pollutants and that increasing pollutant discharge constrained economic growth. Economic growth is the main driving factor that affects the discharge of industrial wastewater pollutants in Hunan Province. On the other hand, the discharge constrains economic growth by producing external pressure on growth, although this feedback mechanism has a lag effect. Economic growth plays an important role in explaining the predicted decomposition of the variance in the discharge of industrial wastewater pollutants, but this discharge contributes less to predictions of the variations in economic growth.

## 1. Introduction

The impact of economic growth on the environment is an emerging and popular issue in resource, environmental, and ecological economics. Resource depletion and pollutant discharge that result from economic growth will inevitably lead to environmental degradation; however, technological progress, economies of scale, and the increased income that result from economic growth can be used to reduce pollutant discharge and ultimately to improve environmental quality. In 1991, Grossman and Kreuger [1] discovered that the relationship between the total discharge of various environmental pollutants (*y*) and economic growth (*x*) takes on the shape of an inverted U-shaped curve. This relationship, which is similar to the relationship between *per capita* income and the distribution level analyzed by the American economist Kuznets, was therefore named the “environmental Kuznets curve” (EKC) [2]. Research on the EKC developed rapidly, reaching a peak during the mid- to late 1990s, when the internal theoretical basis was explored from the perspective of the mechanisms responsible for EKC phenomena.

Since then, many empirical studies have shown that linear, U-shaped, N-shaped, and inverted N-shaped relationships may also exist between the indices of environmental pollution and those of economic growth. Coondoo *et al*. [3] demonstrated the existence of an inverted U-shaped curve for the relationship between gross domestic product (GDP) *per capita* and environmental quality by analyzing panel data from 88 countries and a CO_2_ index. Peng and Bao [4] carried out an empirical analysis of the relationship between GDP *per capita* and multiple environmental pollution indices, and characterized the degree of uncertainty in their relationships using provincial-level panel data for China. This evidence demonstrated that the existing theory required improvement in terms of index characterization, measurement methods, treatment of endogenous defects, variable selection, model improvement, and formation mechanism [5].

The nature of the EKC is that it measures the impact of economic growth on the environment. In terms of the approach to measurement, researchers have developed a range of models based on different assumed conditions and different dominant factors. The most common equation form used in these models is a quadratic equation for the relationship between income and the resource environment. Sometimes an obviously inverted U-shaped curve can be achieved using a quadratic equation of the logarithm to highlight the curve’s characteristic shape. In contrast, an N-shaped cubic equation shows that there are many fluctuations in any real-world system.

Most current models are simple measurements based on single equations, and suggest that the environment has no feedback effect on economic growth. The unidirectional hypothesis, in which the economy influences the environment but the environment does not influence the economy leads directly to inaccurate assessments because it is based on an unrealistic assumption. As Dinda [6] criticized, most researchers have ignored the bidirectional relationship between environmental pollution and economic growth in their investigations of the inverted U-shaped curve for the relationship between environmental and economic parameters. The ignorance of this inverse effect has resulted in the emergence of what is called the “endogeneity bias”, because economic growth itself is an endogenous variable determined by environmental changes and other factors.

Hu *et al*. [7] noted that it is necessary to set up a model that includes the variables that must be endogenized to discuss the interactions between economic growth and environmental quality. Any examination of the relationships among such variables should be carried out with strict and prudent methods, but direct regression analysis may lack a basis for the measurement of endogenous variables. The results of the assessment and their representativeness must also be reconsidered in terms of the lack of data stationarity. Therefore, the improvement of research methods has been a priority for researchers in this area.

There are two main methods to account for endogeneity biases. First, the analysis can be performed by establishing simultaneous equations to produce a dynamic structural formula. For example, Huang and Shaw [8] adopted simultaneous equations in their assessment of the EKC using time-series data from Taiwan. The second method is referred to as a vector auto-regression (VAR) model, and was proposed by Sims as a simplified form of dynamic structural equations and a simpler alternative. In addition, as Lütkepohl [9] pointed out, the advantage of the VAR modeling method is that it provides a good analytical tool for analyzing the dynamic influences of different variables.

The selection of VAR models for use in analyses of the relationships between environmental and economic parameters offers the following advantages: First, there are fewer constraints based on existing theories and assumptions. All the variables in the VAR system are regarded as endogenous and can enter every assessment equation symmetrically. Analysis of the effects of different variables on environmental changes and economic growth would be facilitated in terms of their long-term dynamics, and the problem of variable default would be avoided. Because it is difficult to analyze the economic meaning of test results obtained by direct application of VAR models, the impulse-response function is frequently used for analysis.

In this context, the aim of the present study was to investigate the mutual relationships and interactions between economic growth and the environment (here, using the discharge of industrial waste pollutants as a proxy), and to demonstrate how this analysis could be used in a case study in China’s Hunan Province. We attempted to resolve some of the problems discussed earlier in this paper by using co-integration and error correction models (ECMs). We hope that this study will improve our knowledge of the relationships between economic growth and industrial pollution.

## 2. Methods

### 2.1. The Vector Error Correction Model (VECM)

A VAR model is not based on economic theory, but is instead a regression that investigates the dynamic relationships among all the endogenous variables in a system based on hysteresis effects between changes in the values of each endogenous variable in the model and the resulting changes in the other endogenous variables; that is, there are interactions and path-dependence in these changes. There is no pre-condition (*i.e*., no assumptions) for each estimation process. The function can be described as follows: assuming that **Y***_t_* is a time-sequence vector with a rank of *n* × 1, **Y***_t_* = (*y_1t_*, *y*_2_*_t_*, ···, *y_nt_*)′, where *t* represents time and *y_it_* represents the parameters of the model (from *i* to *n*) at time *t*. The VAR model with rank *k* can be written as:

(1) $$\begin{array}{c} Y_{t}=\Pi_{1}Y_{t-1}+\Pi_{2}Y_{t-2}+\cdots +\Pi_{k}Y_{t-k}+{ɛ}_{t}\;\;\;{ɛ}_{t}∼\text{IID }(0,\Omega )\\ t=1,2,\cdots \cdots ,T \end{array}$$

This function can also be written as VAR (*k*). Π_1_, ···, Π*_k_* are parameter matrices with rank *n* × *n*. ɛ*_t_* is the random-error column vector with rank *n* × 1 and Ω is the variance and covariance matrix with rank *n* × *n*. IID is covariance matrix with rank *n* × *n* and T is time segment.

If **Y***_t_* is not a stable component, the distribution of the parameters in this regression function will not form a normal distribution, and this could result in the misplay of the regular statistical inference [10]. However, Lee and Chang [11] found that if there was a co-integration relationship among the non-stable variables in the VAR model, an autoregression VECM based on the VAR model would make the variables in the VECM become a stationary sequence. In this way, based on equation (1), we can assume that **Y***_t_*∼I in equation (1), and after differential transformation, the VAR model can be expressed as follows:

(2) $$\Delta Y_{\text{t}}=\Gamma_{1}\Delta Y_{t-1}+\Gamma_{2}\Delta Y_{t-2}+\cdots +\Gamma_{k-1}\Delta Y_{t-k}+\Pi Y_{t-k}+{ɛ}_{t}$$

where Δ is the rank difference operator:

$$\begin{array}{l} \Gamma_{1}=-\text{I}-\Pi_{1}-\cdots -\Pi_{i},\;\;\;i=1,2,\cdots ,k\\ \Pi =-\text{I}+\Pi_{1}+\cdots +\Pi_{k} \end{array}$$

Equation (2) is called the regular expression in the VECM. The transformation from equation (1) to equation (2) is called co-integration. The Π in Equation (1) is called the “compression” matrix or the “effect” matrix, and represents the sum of the parameter matrix minus one unit matrix. *i* is the lag order, *k* is the maximum lag order, and Γ is the matrix of coefficients.

The VECM is a type of VAR model, but with a restriction: it includes a co-integration relationship when it explains the variables [12]. When there is a short-term fluctuation over a large range, VECM will make the endogenous variables converge on their long time co-integration relationship. A partial short-term correction is used to correct the departure from the long-term equilibrium. For this reason, the co-integration can also be considered to be an error term.

### 2.2. Data Sources

We adopted the following indicators in our analysis of the discharge of industrial wastewater: the amount of industrial wastewater generated, the amount discharged, the contaminant removal rate, and the amount of pollutants discharged in the industrial waste. Our case study was based on data from Hunan Province in southern China, where annual rainfall is high, and we assumed that the amount of pollutants discharged in industrial waste would have serious effects on the quality of the ecological environment. We adopted the following pollutant parameters (discharge amounts) as proxies for the impact of economic growth on the environment: chemical oxygen demand (COD), ammonia nitrogen (AND), petroleum pollutants (PPD), and heavy metals (HMD).

We obtained time-series data from 1978 to 2007 from the Statistical Yearbook of Hunan Province and the China Environment Yearbook for each year and parameter. We chose two economic growth indicators based on the availability of data and the popularity of these indicators in previous research. On this basis, we chose the *per capita* GDP and the *per capita* consumer price index (CPI), and we analyzed their relative contributions to pollutant discharge by means of principal-components analysis. Both indicators can objectively represent the influence of the economic growth process on industrial wastewater discharge and the resulting pollutant discharge. To eliminate heteroscedasticity and obtain a better fit of the regression, we used the natural logarithm function to transform the data for each parameter.

## 3. Results

### 3.1. Co-Integration Analysis

To make the six variables comparable under the same coordinate, we normalized the values using version 6.0 of the Eviews software (http://www.eviews.com/). All six variables showed a consistent upward trend, but our statistical analysis suggested that the six variables exhibited nonstationarity (Table 1).

To confirm the degree of stability of the sequence, we analyzed the series for each of the six variables independently. We used the augmented Dickey–Fuller test (ADF) for this analysis. The results of the ADF test showed that the first-order difference in the economic environment and the economic growth variables had been taken logarithm. The obvious level was less than the critical value for the ADF unit root test, indicating that the following variables belonged to a first-order integration process I, which means that under the same unit root, the following variables would become a stationary series after single difference.

Co-integration only reflects the long-term dynamic equilibrium among the variables, and the long-term elasticity of the dynamic equilibrium has a positive (or negative) relationship among the variables. The inadequacies of the long-term static model can be compensated for using a correction mechanism when short-term values deviate from the long-term equilibrium, and the correction is implemented using a short-term dynamic model; that is, an error correction model (ECM) can be constructed between the economic and environmental parameters. The error correction can adjust the long-term model for short-term fluctuations in economic growth, and implies that pollution amounts have clear effects on economic growth because of the two-way relationship between economic and environmental parameters. This conclusion must be further confirmed using the Granger causality test.

### 3.2. Determination of Lag Order

To model the parameters with more explanatory power and simultaneously eliminate autocorrelation of the error terms while keeping reasonable freedom, we chose 3 as the maximum lag order. The optimal lag order for a VAR model ranges from level 3 to level 1, in descending order. Akaike’s information criterion (AIC) and the SC information norm and likelihood ratio (LR) statistics were used as the testing standard for choosing the optimum lag order. In addition, we used the *Q* statistic to test whether autocorrelation existed in the remaining sequence, and the White test and ARCH statistics to confirm whether there was heteroscedasticity. For the co-integration test using a multivariate model, we used Johansen’s analytical framework for a VECM (Table 2).

The trace test and the Max-Eigenvalue test of Johansen showed that at *P* < 0.05, there existed a co-integration relationship among the six variables and that there were two co-integration equations (Table 3). The results of this analysis showed that long-term economic growth was negatively correlated with NAD, HMD, PPD, and COD. The long-term elasticity of COD and economic growth was 0.55, which indicated that economic growth in Hunan Province was not strongly sensitive to COD. There was a negative relationship between economic growth and NAD. The long-term fluctuation showed that the long-term elasticity for NAD was 0.77, which means that economic growth was strongly affected by NAD. HMD had the smallest elasticity (0.21) because of changes in Hunan Province’s industrial policy that reduced emissions of heavy metals.

**Figure 1:**
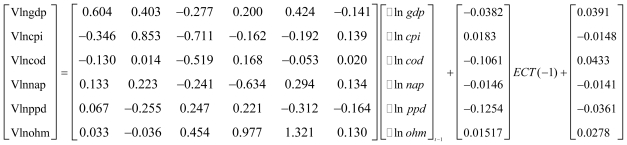
Results of the VECM. *Notes*: GDP: *per capita* GDP; CPI: *per capita* CPI; COD: COD discharge; NAD: nitrogen-ammonia discharge; PPD: petroleum pollutant discharge; HMD: heavy metal discharge.

The VECM had a log-likelihood value of 209.41, which was sufficiently high to provide confidence that the model was reliable. Simultaneously, the AIC and SC values for the VECM were −;16.34 and −;12.64, respectively; both were sufficiently small to indicate that the whole model was well-fitted and had comparatively good explanatory power (Figure 1). After conducting Jarque-Bera multivariate normality tests for the residual error-correction model, we found that the residual error met the requirements for a normal distribution. The serial correlations in the LM and ARCH tests showed that autocorrelation and arch effects did not exist in the model for the residual errors.

### 3.3. Granger Causality Tests

The co-integration among variables only tells us that there is also a long-term causal relationship among the variables. Because the specific direction of the causality (unidirectional or bidirectional) is still unknown, it is necessary to carry out causality tests to define the nature of the relationships among the variables. To do so, we chose the Granger causality test to identify any causality between the indices of economic growth and the pollution discharge indices. The theorem of Engle and Granger states that if there is co-integration among the variables of the VAR model, we can then establish an ECM, which includes error correction terms, and judge the causality among variables based on the VECM. The general form of the VECM, including double variables, is:

(3) $$\Delta {gdp}_{i,t}=\theta_{1,j}+\lambda_{1,iɛi,t-1}+\sum_{k}\theta_{11i,k}\Delta {gdp}_{i,t-k}+\sum_{k}\theta_{izi,k\Delta }{EC}_{i,t-k}+u_{1i,t}$$

(4) $$\Delta {EC}_{i,t}=\theta_{2,j}+\lambda_{2,iɛi,t-1}+\sum_{k}\theta_{21i,k}\Delta {gdp}_{i,t-k}+\sum_{k}\theta_{22i,k\Delta }{EC}_{i,t-k}+u_{2i,t}$$

where Δ denotes the D-value, *k* denotes the lag phase, *EC*_1,_*_t_*_−1_ indicates the first-order lag residuals (error-correction terms) in the co-integration test results, and the causality can be determined by testing the significance of the independent variation coefficients in Equations (3) and (4). For the short-term causality, we can test all *i* and *k* using the null hypothesis H_0_:θ_12_*_ik_* = 0 in Equation (3) for all *i* and *k* and H_0_:θ_21_*_ik_* = 0 in Equation (4). The long-term causality can then be tested by observing the significance of the modification of rate λ, with λ as the coefficient of the error correction form ɛ*_it_*_-1_. The significance of λ is that it reflects the long-term relationships identified by the co-integration process. The coefficients of the EC term contain information on whether the past variable values affect the current values. A significant non-zero coefficient indicates that error terms in the past equilibrium play an important role in determining the current results. For the long-term causality, we tested all *i* for the null hypothesis H_0_:λ_1_*_i_* = 0 for all *i* and *k* in equation (3) and for all *i* for the null hypothesis H_0_:λ_2_*_i_* = 0 in equation (4).

We draw the following conclusions from the test results in Table 4: economic growth is an important cause of changes in environmental quality. For COD, the short-term causality values deduced from Equations (3) and (4) are statistically significant (*P* < 0.05), which shows an obvious short-term causality between COD and GDP and a bidirectional relationship between COD and GDP in the long term. For NAD, the short-term causality deduced from Equations (3) and (4) is significant (*P* < 0.05), which shows obvious bidirectional causality between NAD and GDP in the short term and a bidirectional causality in NAD in the long term. There is no obvious causality between PPD and GDP in the short term, but there is a bidirectional causality between PPD and GDP in the long term. For HMD, the short-term causality deduced from Equations (3) and (4) was significant (*P* < 0.05), which shows an obvious bidirectional causality between HMD and GDP in the short term; moreover, there is bidirectional causality in NAD in the long term. Although there is no obvious causality between PPD and GDP, the Granger tests show that changes in GDP can cause changes in the discharge of the four pollutants.

### 3.4. Impulse Response Function for Economic Growth and Environmental Pollution

**Figure 2:**
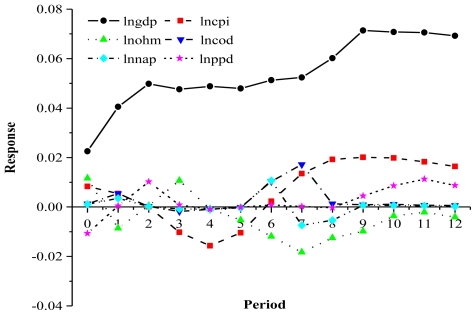
Impulse responses of GDP and CPI and of the four pollutant discharges. *Notes*: GDP: *per capita* GDP; CPI: *per capita* CPI; COD: COD discharge; NAD: nitrogen-ammonia discharge; PPD: petroleum pollutant discharge; HMD: heavy metal discharge.

The impulse response function describes the response of an endogenous variable to the impact of error terms. The impulse response function based on the VAR model can be used to measure the impact of a change of 1 standard deviation in one variable that results from stochastic disturbance on the current and future values of the other variables, and can be used to analyze the whole process of how a disturbance of any variable in the VAR model will influence the other variables and ultimately the variable itself. We used general impulse response analysis in this part of our analysis. Its main function is to provide a more prudent scheme for the orthogonalization process than in the traditional impulse response analysis, and its main value is that it can provide a more prudent conclusion based on orthogonality of the impulses and no connection with the order in which the variables are arranged. Figure 2 shows the first-order differences in the normalized, ln-transformed values of the model parameters.

During the whole impulse response period, the response curve for COD to a one-unit response of GDP was N-shaped: the response value of COD in terms 1 and 2 was positive, decreased towards zero in terms 3 to 5, became positive again in terms 5 to 8, then finally decreased to remain around zero from terms 8 to 12. This suggests that the overall influence of GDP on COD was to increase this discharge, and the cumulative response was 0.186, which indicates that economic growth increases COD.

The impulse response for NAD approximately resembled an N-shaped curve. The response value in terms 1 to 3 was positive, decreased towards zero from terms 3 to 5, was positive in term 6, was negative in terms 7 and 8, and then remained near zero from term 9 to 12. The impulse response of NAD to GDP was generally an increase in NAD, and the cumulative response was 0.034. The economic meaning of this result is that economic growth would increase NAD and that increased NAD would slow the rate of economic growth.

The impulse response of PPD to GDP also took on an N-shaped curve. The response value of PPD during terms 1 to 3 changed from negative to positive, decreased to remain near zero in terms 3 to 8, and then became positive again from terms 9 to 12. The cumulative response was 0.027. Economic growth would therefore increase PPD.

The impulse response of HMD to GDP took on a double U-shaped curve. The response values of HMD to GDP were initially positive then decreased to become negative in term 1, then increased to become positive in term 3 (the first U), then decreased from term 3 to term 7 and increased again to near zero by term 10 (the second U); thereafter, it remained stable near zero. The cumulative response was 0.215, which indicates that economic growth increases HMD.

The response of GDP to pollutant discharge took on an inverted U-shaped curve. This demonstrates the adverse effects of environmental degradation and pollutant discharge on economic growth: As environmental quality deteriorated and pollutant discharge increased, changes in human preferences related to environmental quality and modulation of the industrial structures imposed external pressure on the mode of economic growth. At the beginning of economic growth, the effects of pollutant discharge on economic growth were small. Therefore, during the first three terms, the GDP curve increased rapidly, and all pollutant discharge response values reached their maximum value, step by step. With continuous economic growth, the discharge slowly became a constraint on economic growth and slowed the rate of economic growth. Thus, when GDP was close to a certain saturated level, during the third to sixth term, the response changed to a nearly horizontal line. From the eighth term onwards, the response of GDP to waste discharge was to decrease, which means that waste discharge became a significant constraint on economic expansion, a constraint on economic expansion until wastewater treatment capacity increased. The contradiction between economic growth and waste discharge then moderated, and GDP once more grew at a good pace, until a new round of adjustment began.

### 3.5. Variance Decomposition Based on VECM

Decomposition of variance refers to calculation of the mean square error of the impact of a variable into the contribution created by a random impulse from various variables, followed by calculation of the relative importance of each variable’s impulse (*i.e*., its contribution to the total variance). The decomposition therefore quantitatively captures the relationship among the variables in the VAR model.

**Figure 3:**
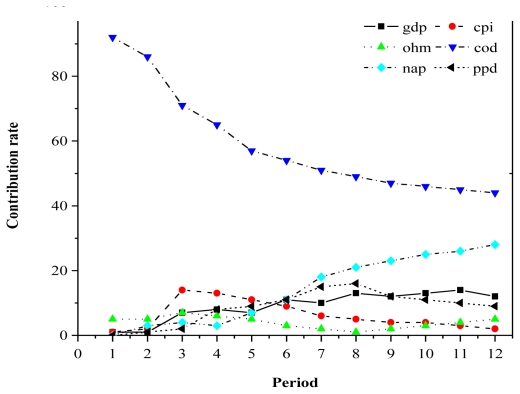
The decomposition of variance for GDP. *Notes*: GDP: *per capita* GDP; CPI: *per capita* CPI; COD: COD pollutant discharge; NAD: nitrogen-ammonia discharge; PPD: petroleum pollutant discharge; HMD: heavy metal discharge.

Because of space constraints, we will use decomposition of variance for *per capita* GDP and COD to illustrate only two typical examples of variance decomposition. Figure 3 shows that after the term 4, NAD increased continuously, and was increasingly affected by the *per capita* GDP, and eventually accounted for more than 20% of the predicted variance in *per capita* GDP by term 8, which indicates that the long-term influence of NAD on *per capita* GDP was increasing, and gradually became an important factor that slowed economic growth. The effects of NAD on *per capita* GDP gradually decreased (*i.e.*, the GDP curve appeared to be approaching an asymptote), accounting for 73% of the predicted variance, and then remained stable, indicating that NAD did not have a remarkable influence on economic growth in the short term, but had a remarkable influence in the long term.

**Figure 4:**
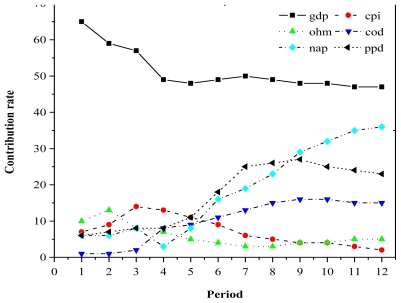
The decomposition of variance for COD. *Notes*: GDP: *per capita* GDP; CPI: *per capita* CPI; COD: COD pollutant discharge; NAD: nitrogen-ammonia discharge; PPD: petroleum pollutant discharge; HMD: heavy metal discharge.

Figure 4 shows that the predicted variance for COD mainly resulted from the effects of economic growth and itself. The effects of COD on economic growth accounted for about 57% of the predicted variance in COD, which indicates a strong influence on economic growth. The change in COD was obviously towards a weaker trend over time (*i.e.*, the curve reached a plateau at around 15%), with the proportion of the predicted variance decreasing to 28.7%. The predicted variance for *per capita* GDP during terms 1 to 3 was weakening, but reached 2.8%; from term 4 onwards, its effects increased more rapidly, finally reaching a value of around 15% by term 12.

## 4. Discussion

Co-integration relationships represent a kind of long-term statistical interpretation that balances the relationships among non-stable variables. The co-integration represents a certain linear combination of pairs of non-stable variables, and offers a certain degree of stability. The co-integration analysis tests whether there is a stable linear combination of relationships among non-stable variables and seeks a co-integrated relationship among the variables [13]. This form of analysis is necessary because applying direct regression analysis to a non-stationary time series can cause problems such as a spurious regression result. It is necessary to begin by judging the stationarity of the time series. This is tested using DF or ADF to conduct the unit root test [14]. The DF test assumes that the random error term of the tested models has no autocorrelation; however, most economic data series cannot meet this assumption [15].

We used the Jarque-Bera test for normality of the residual error because our analysis showed that when the remaining sequence of each regression equation met the condition of normality (*P* < 0.05), this indicates a lack of autocorrelation and heteroscedasticity [16]. Therefore, the goodness-of-fit of the VAR model with a lag order of 1 was quite good, and the remaining sequence exhibited stability. Maki [17] noted that time-series data such as those used in this paper may not be stable and are likely to be affected by common factors, and would therefore reflect a common trend over time. This would mean that a stability relationship existed among the variables and that some linear combination of the variables would be stable. The co-integration relationship made it clear that a long-term and balanced relationship existed among the variables in our study. This relationship was realized through constant adjustment of the error-correction terms to avoid serious errors in the long-term balanced relationship [18].

Under normal circumstances, with more than one co-integration relationship among the variables, the first co-integration equation correctly reflects the long-term relationship among the variables [19]. Bekiros and Diks [20] stated that according to Granger’s typical theorem, a set of variables for which a co-integration relationship existed could be used to establish an error-correction model. If a co-integration relationship existed among these variables, this would mean that a long-term stable relationship existed among these variables and was maintained through constant adjustment of short-term dynamic processes. This error-correction mechanism avoided expansion of the deviation for the long-term relationship in terms of both quantity and scope. Thus, an error-correction mechanism existed in the time series for the study variables, which was inter-coordinated to produce short-term adjustment behavior. Based on the error-correction model, the short-term dynamic relationship among these variables and the error correction model were established [21].

An impulse response function based on the VAR model can be used to measure the effects of the impact of a one-standard-deviation random disturbance term on all the current and future values of the variables. This function can be used to analyze how a disturbance of any variable in the VAR model would affect other variables in the model, finally resulting in feedback that affects the variable itself [22]. It is therefore natural that there would be a negative effect of increased HMD on economic growth. The reason for this may be that it took a long time for environmental technology to adjust to the industrial structure. The basic principle of variance decomposition is to decompose the predicted mean-square error for any of the endogenous variable into the contribution of the random impacts to the overall system. Calculating the comparative importance of each variable to determine its proportional contribution to the overall system variation accounted for the whole contribution [23]. Variance decomposition can be used to analyze the contribution of each variable, and therefore, reflected the relative importance of random impacts of each variable on the VAR systematic variable [24].

In general, the decomposition result for comprehensive variance showed that economic growth played an important role in explaining various proportions of the predicted variance based on pollutant discharge indicators. Economic growth caused a predicted variance for COD, NAD, PPD, and HMD that together exceeded 72%. The results showed that economic growth in Hunan Province accompanied by increased wastewater discharge became an important factor in damage to the ecological environment. Comparatively speaking, the discharge of industrial wastewater accounted for a relatively small impact on economic growth. The contribution of COD was only 16.8%, which was much lower than that of the discharge of industrial wastewater. The influences of NAD and PPD were also relatively weak. One reason for this may be that as a result of maturation of Hunan Province’s economic development, the province increased its pollution control measures and its treatment of NAD and PPD. However, another possible reason is that during the process of data calculation, statisticians may make the statistical data lower than the actual due to subjective differences or governmental directives.

## 5. Conclusions

We have conducted a dynamic econometric analysis of Hunan Province’s economic growth and its discharge of industrial wastewater based on time-series data from 1978 to 2007. To do so, we used a co-integration test, impulse response analysis, and variance decomposition. The results showed a long-term balance between economic growth and the discharge of industrial wastewater. Active and positive effects of economic growth on this discharge and a feedback effect of wastewater discharge on economic growth were both clearly revealed.

## Figures and Tables

**Table 1:** Results of the unit root tests.

Variance	Test method	ADF test value	Critical value	Critical value	Conclusion
ΔlnGDP	(C,T,0)	−3.1593	−2.9762	−3.6998	stable
ΔlnCPI	(C,T,0)	−2.6692	−2.6299	−2.9810	stable
ΔlnCOD	(C,T,0)	−6.0544	−2.9763	−3.6998	stable
ΔlnNAD	(C,T,0)	−4.8663	−2.9762	−3.6998	stable
ΔlnPPD	(C,T,0)	−6.4657	−2.9762	−3.6998	stable
ΔlnHMD	(C,T,0)	−3.7614	−2.9762	−3.6998	stable

*Notes*: C: constant term; T: tendency; 0: lag order; Δ: first-order difference; GDP: *per capita* GDP; CPI: *per capita* CPI; COD: COD discharge; NAD: nitrogen-ammonia discharge; PPD: petroleum pollutant discharge; HMD: heavy metal discharge.

**Table 2:** Results of the Johansen test for co-integration.

No. of CEs	Trace statistic	Trace test		Max-Egon	Max-Egon test	
		5% critical value	Prob		5% critical value	prob
None	132.7742	95.7536	0.0000	55.5041	40.0776	0.0005
At most 1	77.2701	69.8189	0.0113	35.5217	33.8769	0.0316
At most 2	41.7484	47.8561	0.5934	24.1177	27.5843	0.1307
At most 3	17.6306	29.7971	0.6736	11.4452	21.1316	0.6029

**Table 3:** Normalized co-integration equations.

GDP	CPI	COD	NAD	PPD	HMD
1.0000	−1.0297	0.5513	0.7715	0.2110	0.2107
	0.2010	0.4655	0.0128	0.4314	0.6138

*Notes*: GDP: *per capita* GDP; CPI: *per capita* CPI; COD: COD discharge; NAD: nitrogen-ammonia discharge; PPD: petroleum pollutant discharge; HMD: heavy metal discharge.

**Table 4:** Results of the Granger causality tests.

Pollutant	Lag order	Short term		Long term	

		H_0_: GDP does not Granger cause ED	H_0_: ED does not Granger cause GDP	H_0_: GDP does not Granger cause ED	H_0_: ED does not Granger cause GDP
COD	4	4.596 (0.061)	4.403 (0.828)	6.894 (0.049)	3.408 (0.433)
NAP	5	2.891 (0.426)	3.363 (0.848)	4.369 (0.317)	3.612 (0.249)
PPD	3	4.635 (0.158)	5.452 (0.719)	3.312 (0.543)	2.612 (0.342)
HMD	3	5.159 (0.023)	4.605 (0.256)	5.894 (0.249)	3.408 (0.437)

*Notes*: ED (environmental discharge) represents the logarithm of the values of the four environmental pollution variables; GDP: *per capita* GDP; COD: COD discharge; NAD: nitrogen-ammonia discharge; PPD: petroleum pollutant discharge; HMD: heavy metal discharge.
